# Global Leadership Initiative on Malnutrition: a bibliometric analysis of research trends and contributions (2018–2024)

**DOI:** 10.3389/fnut.2025.1613395

**Published:** 2025-07-08

**Authors:** Qimeng Xu, Qiufu Li, Yucheng Yao, Ying He, Sitao Tan, Xiaoxia Liu, Xiaoyu Chen

**Affiliations:** ^1^Department of Pharmacy, Guangxi Academy of Medical Sciences and the People’s Hospital of Guangxi Zhuang Autonomous Region, Nanning, China; ^2^Department of Pharmacy, Guilin Medical University, Guilin, China

**Keywords:** GLIM criteria, malnutrition, bibliometric analysis, sarcopenia, nutritional assessment, clinical application

## Abstract

**Background:**

The Global Leadership Initiative on Malnutrition (GLIM) criteria were officially introduced in 2018 with the aim of establishing a standardized global framework for the diagnosis of malnutrition. Synthesizing expert consensus from multiple international organizations, the GLIM criteria proposed a two-step diagnostic model integrating both phenotypic and etiologic components. Although GLIM-related research has expanded rapidly in recent years, a comprehensive bibliometric evaluation remains absent.

**Methods:**

Relevant literature published between 2018 and 2024 was retrieved from the Scopus database. Only English-language original research articles and reviews were included. A total of 729 eligible publications were analyzed using VOSviewer (v1.6.10), CiteSpace (v5.8.R3), and the online platform Bioinformatics. The analysis covered various dimensions, including countries, institutions, authors, journals, keywords, and highly cited references.

**Results:**

The volume of GLIM-related publications has shown a steady upward trajectory, peaking in 2024. China emerged as the most prolific country, followed by Spain and Japan. The top contributing institutions included Uppsala University, Capital Medical University, and Beijing Shijitan Hospital. Among the most productive authors were Cederholm T, Shi H, and Correia MITD. *Clinical Nutrition* and *Nutrients* were identified as the core journals in this field. Keyword analysis revealed that “malnutrition,” “diagnosis,” “sarcopenia,” “cancer,” and “nutritional risk” were pre-dominant themes, while “systematic review,” “protein blood level,” and “gastric cancer” represented emerging areas of interest.

**Conclusion:**

This study represents the first comprehensive bibliometric analysis of research related to the GLIM criteria. It identifies key contributors, collaboration networks, and thematic evolutions in the field, highlighting a transition from the development of diagnostic frameworks to clinical application and individualized nutritional assessment. These findings provide a valuable reference for guiding future research directions in GLIM-related domains.

## 1 Introduction

Malnutrition remains a significant global health concern, adversely affecting patient outcomes and straining healthcare systems through increased risk of complications, prolonged hospital stays, higher readmission rates, elevated mortality, and greater medical expenditures (1–4). Current diagnostic approaches face challenges in standardization, as conventional indicators—such as dietary intake, weight changes, and serum albumin levels—have inherent limitations that preclude their use as standalone diagnostic criteria (5–7). To address these limitations, various international nutritional societies have proposed different diagnostic frameworks. In 2012, the American Society for Parenteral and Enteral Nutrition (ASPEN) and the Academy of Nutrition and Dietetics (AND) jointly introduced a two-step diagnostic algorithm incorporating both etiologic and phenotypic components. In contrast, the 2015 consensus by the European Society for Clinical Nutrition and Metabolism (ESPEN) emphasized body mass index (BMI) as a core parameter (5, 8). These discrepancies underscore the pressing need for a globally unified diagnostic standard for malnutrition. In 2018, the Global Leadership Initiative on Malnutrition (GLIM) proposed a standardized diagnostic framework that combines phenotypic criteria (involuntary weight loss, low BMI, and reduced muscle mass) with etiologic criteria (reduced food intake/assimilation and inflammation/disease burden) (9). A diagnosis of malnutrition under the GLIM criteria requires the presence of at least one phenotypic and one etiologic criterion. This framework offers a practical and universally applicable diagnostic model adaptable to various populations and clinical contexts, thereby advancing the global standardization of malnutrition assessment. The specific diagnostic components of the GLIM framework are summarized in Table 1.

**TABLE 1:** Diagnostic criteria and thresholds of the GLIM.

Criteria category	Diagnostic criteria	Specific details
Phenotypic criteria	Unintentional weight loss	Weight loss > 5% within 6 months, or > 10% over > 6 months
	Low BMI	General population: Age < 70 years: BMI < 20 Age ≥ 70 years: BMI < 22
		Asian population (regional guidelines): Adults: BMI < 18.5 Age ≥ 70 years: BMI < 20 (per some guidelines)
	Reduced muscle mass	Primary methods: Body composition analysis (e.g., DXA, BIA, CT, MRI)
		Supportive methods: Grip strength (men < 27 kg, women < 16 kg), calf circumference (< 31 cm), skinfold thickness
Etiologic criteria	Reduced food intake/absorption	Energy intake < 50% of requirements for ≥ 1 week; or chronic gastrointestinal disorders impairing intake/absorption (e.g., diarrhea, dysphagia)
	Inflammation/disease burden	Acute disease/trauma (e.g., infection, surgery) Chronic disease-related inflammation (e.g., cancer, COPD, heart failure)
		Supportive marker: CRP > 5 mg/L (indicating inflammatory activity)

A diagnosis of malnutrition requires the presence of at least one phenotypic criterion and one etiologic criterion. BMI, Body Mass Index; DXA, Dual-energy X-ray Absorptiometry; BIA, Bioelectrical Impedance Analysis; CT, Computed Tomography; MRI, Magnetic Resonance Imaging; CRP, C-reactive Protein; COPD, Chronic Obstructive Pulmonary Disease; ESPEN, European Society for Clinical Nutrition and Metabolism; GLIM, Global Leadership Initiative on Malnutrition.

Bibliometrics is a methodological approach that quantitatively and qualitatively analyzes scientific literature to assess publication output and research trends within a given field (10, 11). It enables the extraction and synthesis of information on authors, keywords, journals, countries, institutions, and references (11). Widely used bibliometric tools such as CiteSpace (12), VOSviewer (13), the R package bibliometrix (14), and HistCite (11) facilitate visualization and interpretation of analytical data, and have been extensively applied in medical disciplines, including oncology (15, 16), orthopedics (17), thoracic surgery (18), and rheumatology (19). As a globally endorsed framework, the GLIM criteria have played a pivotal role in establishing standardized diagnostic protocols and guiding clinical nutrition interventions. In recent years, the number of publications related to GLIM has steadily increased; however, systematic evaluations and methodological syntheses remain relatively scarce. Therefore, conducting a comprehensive bibliometric analysis is both timely and necessary. This study aims to systematically analyze the literature on GLIM from 2018 to 2024, with the objectives of identifying influential contributors, mapping the evolution of research priorities, and highlighting future directions for the development and refinement of GLIM standards.

## 2 Methods

### 2.1 Data sources and search strategy

All relevant publications were retrieved from the Scopus database, which offers a comprehensive and standardized export format widely used in academic research. To minimize potential bias caused by continuous database updates, the search was conducted on a single day (February 17, 2025). The search strategy was as follows:

[TITLE-ABS-KEY(global AND leadership AND initiative AND on AND malnutrition) OR TITLE-ABS-KEY(glim)].

**FIGURE 1:**
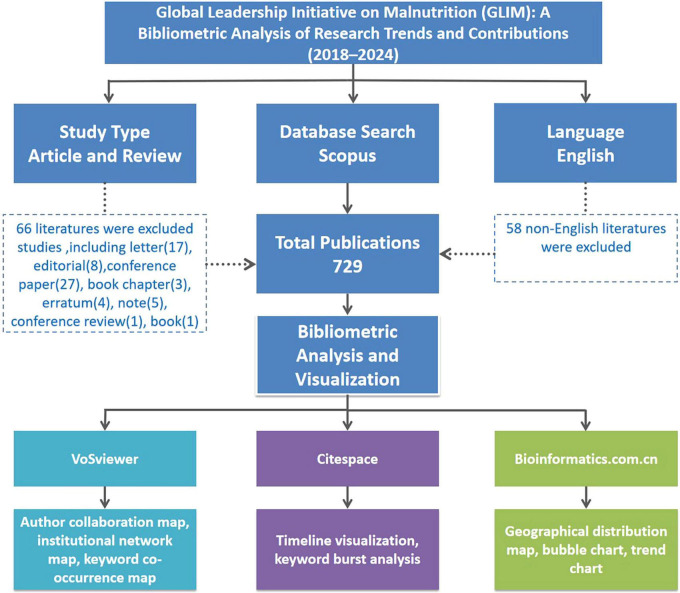
Flowchart illustrating the literature selection process and bibliometric analysis framework for GLIM-related research from 2018 to 2024. A total of 729 English-language articles and reviews were identified from Scopus. Non-English publications and other non-eligible document types were excluded. Bibliometric visualization was conducted using VOSviewer, CiteSpace, and the Bioinformatics online platform.

The search was restricted to English-language original research articles and reviews published between 2018 and 2024. A total of 729 eligible records were initially identified. The detailed screening process is illustrated in Figure 1.

### 2.2 Data collection

The identified records were exported and processed using Microsoft Excel 2016. Extracted bibliographic information included authors, affiliations, countries/regions, journals, number of publications (Np), number of citations (Nc), publication years, H-index, keywords, and references. The data were then used for further bibliometric analysis, and the original dataset has been provided as Supplementary material to support transparency and reproducibility.

### 2.3 Bibliometric analysis

Bibliometric analysis was performed using VOSviewer (version 1.6.10), CiteSpace (version 5.8.R3), and the online platform.^1^ Two primary indicators—number of publications (Np) and number of citations (Nc)—were used to assess research performance. Np reflects scientific productivity, while Nc indicates academic influence. Both are standard measures for evaluating the value of scholarly output. The H-index was used to evaluate individual academic contributions and potential future impact. An H-index of h indicates that a researcher has published h papers, each cited at least h times. While initially intended for individual evaluation, the H-index is now also used to measure the scholarly performance of countries, institutions, and journals (20, 21).

The Impact Factor (IF), as reported in the latest Journal Citation Reports (JCR), remains one of the most recognized metrics for evaluating journal quality and influence in the medical field (22). The Global Citation Score (GCS) refers to the total number of citations an article has received worldwide and serves as an important indicator of its contribution to the scientific community (23). To model the temporal trend of research output, a polynomial regression model was fitted to the annual number of publications. In this model, the number of publications per year is represented by f(x), with x denoting the publication year.

VOSviewer (version 1.6.10.0) was used to construct and visualize bibliometric network maps, including co-citation and keyword co-occurrence analyses (24, 25). In the resulting visualizations, node size represents the number of publications, line thickness reflects the strength of relationships, and different colors are used to distinguish clusters or time periods.

CiteSpace (version 5.8.R3) was applied to explore research hotspots and thematic evolution through keyword clustering, timeline views, and burst detection analysis (26). Keyword clustering helps delineate research themes and identify core topics in GLIM-related studies. The timeline view visualizes the temporal evolution of keyword clusters, while burst detection reveals emerging topics that received sudden attention during specific time intervals.

The Bioinformatics online platform^2^ supports the generation of various graphical modules, such as geographical distribution maps, bubble charts, and trend plots. With a user-friendly graphical interface, it allows researchers without programing experience to produce high-quality, publication-ready figures (exportable in PDF or SVG formats), and is widely used in bibliometrics and bioinformatics research (27).

## 3 Results

### 3.1 Overview of GLIM-related publications

**FIGURE 2:**
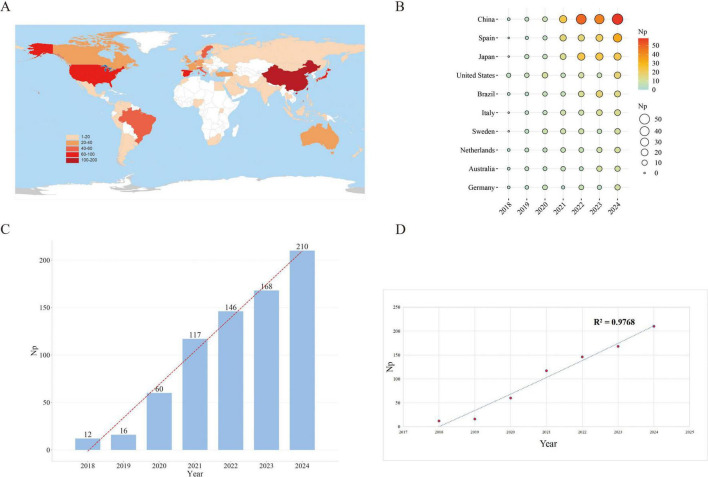
Trends in GLIM-related publication output from 2018 to 2024. **(A)** Global distribution of GLIM-related publications by country. **(B)** Annual publication output of the top 10 contributing countries, with circle size and color intensity reflecting publication volume. **(C)** Yearly number of publications, illustrating a steady increase over time. **(D)** Polynomial regression curve indicating the overall growth trend, with an *R*^2^ of 0.9768. Np, Number of publications.

A total of 729 GLIM-related publications were retrieved from the Scopus database between 2018 and 2024, including 683 original research articles and 46 reviews. These publications received a total of 13,146 citations (Nc), with an average of 18.03 citations per article. The collective H-index for all publications was 49. The global distribution of GLIM-related research is shown in Figure 2A. The top five contributing countries—China, Spain, Japan, the United States, and Brazil—accounted for approximately two-thirds of the total output, revealing notable regional disparities in research activity.

Among the top 10 countries contributing annually (Figure 2B), China consistently ranked first, publishing 58 papers in 2024 alone, followed by Spain (33) and Japan (22). China’s consistently high output underscores its leading role in this field, as well as the nation’s sustained academic investment and strategic prioritization of GLIM research.

### 3.2 Annual trend in publication volume

Figure 2C illustrates the yearly number of GLIM-related publications. The volume of publications increased markedly from 12 in 2018 to 210 in 2024, with Np reaching its historical peak in 2024. This growth trajectory indicates that GLIM has gradually emerged as a central topic in global academic research, with research activity exhibiting a trend of exponential growth.

Figure 2D displays the linear regression curve (dashed line) representing the year-by-year change in publication output (*R*^2^ = 0.9768). Although minor fluctuations were observed in certain years, the overall trend demonstrates a nearly linear and robust upward trajectory. Notably, since 2020, the annual growth rate has accelerated further, highlighting the accelerating academic interest and cumulative momentum in GLIM-related research.

### 3.3 National contributions to global publication output

The top 10 countries/regions with the highest academic output were identified (Table 2). China ranked first with 191 publications, accounting for 26.20% of the total (729), followed by Spain (93, 12.76%) and Japan (92, 12.62%). In terms of Nc, China led with 5,335 citations, followed by Italy (4,188), Japan (4,154), and the United States (4,143). China also had the highest H-index (26), followed by Spain (22), Japan (20), and a four-way tie among the United States, Italy, Sweden, and the Netherlands (all at 20). Notably, although Spain ranked second in publication volume (Np = 93), its citation count (1,790) was substantially lower than those of countries with similar Np, such as Japan (4,154) and the United States (4,143)—less than half in both cases. However, Spain’s H-index (22) was second only to China (26) and exceeded that of Japan (20).

**TABLE 2:** Top 10 countries/regions in GLIM-related research output (2018–2024).

Rank	Country/region	Np	% of (729)	Nc	H-index
1	China	191	26.20%	5,335	26
2	Spain	93	12.76%	1,790	22
3	Japan	92	12.62%	4,154	20
4	United States	62	8.50%	4,143	20
5	Brazil	59	8.09%	4,021	19
6	Italy	51	7.00%	4,188	20
7	Sweden	47	6.45%	4,110	20
8	Netherlands	43	5.90%	3,904	20
9	Australia	39	5.35%	3,757	18
10	Germany	35	4.80%	4,032	17

Np, number of publications;% of (729), percentage of total publications (729); Nc, total number of citations; H-index, metric combining productivity and citation impact.

### 3.4 Analysis of affiliations

Affiliation-level analysis was conducted to identify the top 10 institutions in terms of GLIM-related publication output. As shown in Table 3, Capital Medical University (China) had the highest number of publications (Np = 36), followed by Uppsala University (Sweden, Np = 35) and Beijing Shijitan Hospital (China, Np = 33). Citation performance and academic impact were evaluated using total citation count (Nc) and H-index, revealing notable regional variations. Uppsala University ranked first with an Nc of 4,024 and an H-index of 19. Karolinska University Hospital (Sweden) and the Federal University of Minas Gerais (Brazil) followed with Nc values of 3,719 and 3,682, respectively. While six of the top 10 institutions were based in China, including Capital Medical University, Beijing Shijitan Hospital, and the Chinese Academy of Medical Sciences & Peking Union Medical College, these institutions generally showed lower Nc values (e.g., 1,003 and 684, respectively) and H-indices (ranging from 12 to 17). The Carlos III Institute of Health (Spain) had the lowest Nc among the top 10 institutions (299). These metrics collectively reflect institutional differences in research productivity and impact within the GLIM field.

**TABLE 3:** Top 10 affiliations in GLIM-related research based on publication output and citation impact (2018–2024).

Rank	Affiliations	Country	Np	Nc	H-index
1	Capital Medical University	China	36	1,003	17
2	Uppsala University	Sweden	35	4,024	19
3	Beijing Shijitan Hospital	China	33	979	17
4	Chinese Academy of Medical Sciences & Peking Union Medical College	China	30	684	13
5	Karolinska University Hospital	Sweden	27	3,719	17
6	Carlos III Institute of Health	Spain	23	299	10
7	Army Medical University	China	23	713	15
8	Zhengzhou University	China	22	510	14
9	Federal University of Minas Gerais	Brazil	22	3,682	15
10	The First Bethune Hospital of Jilin University	China	22	610	12

Np, Number of publications; Nc, Total number of citations; H-index, Metric combining research productivity and citation impact.

### 3.5 Author analysis

**FIGURE 3:**
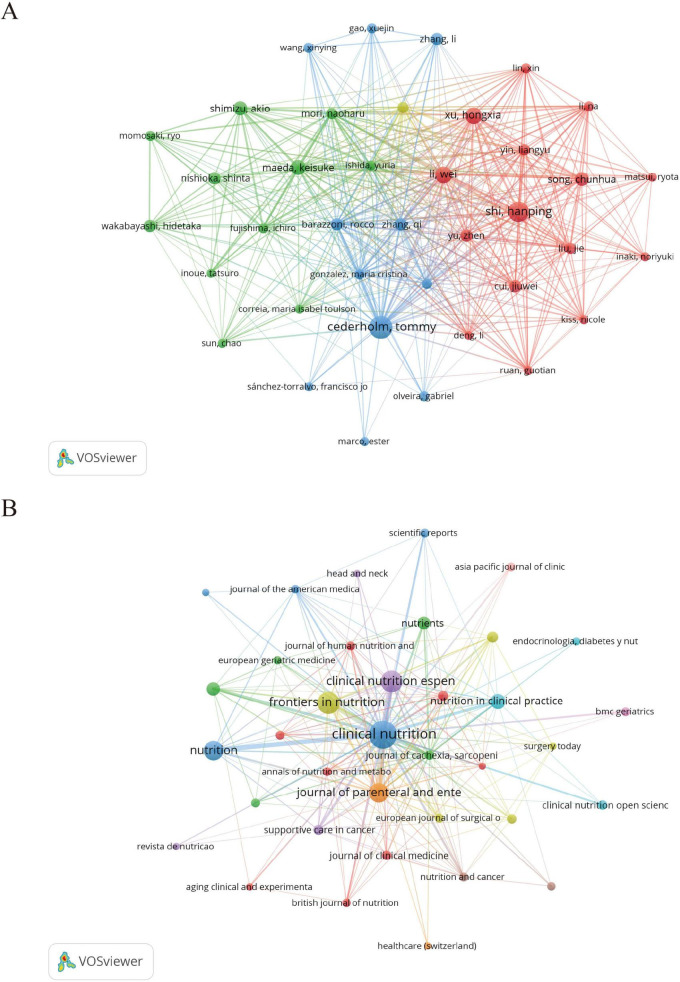
Author and journal co-citation network maps generated by VOSviewer. **(A)** Author co-citation network based on 729 GLIM-related publications. A minimum citation threshold of 7 was applied, resulting in 38 authors included in the analysis. **(B)** Journal co-citation network with a minimum citation threshold of 3, including 37 journals. In both maps, node size represents citation frequency, node color indicates cluster grouping, and line thickness reflects the strength of co-citation links.

Table 4 lists the top 10 most prolific authors in the field of GLIM research. To further explore their academic influence and collaborative patterns, an author co-citation network was constructed (Figure 3A) to identify highly cited researchers and their interconnections within the field. Collectively, these authors contributed 178 publications, accounting for 24.4% of the total global output in this domain. Among them, Cederholm, T. from Uppsala University (Sweden) ranked first with 30 publications, 3,937 citations (Nc), and an H-index of 18. His work has exerted substantial influence on the development and validation of the GLIM criteria. In addition, Correia, M.I.T.D. from the Federal University of Minas Gerais (Brazil) and Barazzoni, R. from the University of Trieste (Italy) exhibited significant impact in the clinical application of the GLIM framework, with Nc values of 3,667 and 3,782, respectively. From a regional perspective, Chinese scholars were highly productive, occupying five of the top 10 positions. For example, Shi, H. from Beijing Shijitan Hospital (Nc = 422) and Xu, H. from Army Medical University (Nc = 491) made notable contributions to the clinical validation and dissemination of the GLIM criteria in China. However, despite their high publication output, Chinese researchers exhibited relatively lower citation counts. This may be related to certain gaps in theoretical innovation and international academic influence. In contrast, scholars from Europe and the United States demonstrated greater citation impact, indicating higher recognition of their work in both academic and clinical contexts.

**TABLE 4:** Top 10 most productive authors in GLIM-related research (2018–2024).

Rank	Author	Affiliations	Country	Np	Nc	H-index
1	Cederholm, T.	Uppsala University	Sweden	30	3,937	18
2	Shi, H.	Beijing Shijitan Hospital	China	22	422	12
3	Correia, M.I.T.D.	Universidade Federal de Minas Gerais	Brazil	19	3,667	14
4	Xu, H.	Army Medical University	China	18	491	13
5	Li, W.	The First Bethune Hospital of Jilin University	China	17	555	11
6	Barazzoni, R.	University of Trieste	Italy	17	3,782	16
7	Gonzalez, M.C.	Federal University of Pelotas	Brazil	16	3,594	14
8	Song, C.	Zhengzhou University	China	15	377	10
9	Yin, L.	Chongqing Southwest Hospital	China	12	425	11
10	Shimizu, A.	Mie University	Japan	12	233	10

Np, Number of publications; Nc, Total number of citations; H-index, Metric combining research productivity and citation impact.

### 3.6 Journal analysis

As shown in Table 5, *Nutrients* ranked first in terms of publication volume on GLIM-related research, with 98 articles published [impact factor (IF) = 4.8]. It was followed by *Clinical Nutrition*, which published 76 articles (IF = 6.6) and exhibited the highest academic impact in this field, with the greatest number of citations (Nc = 4,394) and the highest H-index (29) among the top 10 journals. *Frontiers in Nutrition* ranked third, with 44 publications (IF = 4.0).

**TABLE 5:** Top 10 journals in GLIM-related research by publication volume (2018–2024).

Rank	Journal	Np	Nc	H-index	IF(2023)
1	Nutrients	98	1,915	20	4.8
2	Clinical Nutrition	76	4,394	29	6.6
3	Frontiers in Nutrition	44	306	10	4.0
4	Clinical Nutrition ESPEN	41	430	10	2.9
5	Journal of Parenteral and Enteral Nutrition	33	1,222	16	3.2
6	Nutrition	32	516	12	3.2
7	Nutrition in Clinical Practice	17	119	6	2.1
8	Journal of Nutrition	13	175	8	3.7
9	European Journal of Clinical Nutrition	9	120	7	3.6
10	Current Opinion in Clinical Nutrition and Metabolic Care	7	125	5	3.0

Np, Number of publications; Nc, Total number of citations; H-index, Metric combining research productivity and citation impact; IF(2023), 2023 Journal Impact Factor.

To further explore core journals and their inter-citation relationships, a journal co-citation network was constructed (Figure 3B). The top 10 journals contributed 370 publications, representing 50.75% of the total output (370/729), underscoring their dominant role in the GLIM research landscape. Notably, among the top 10 journals, only *Clinical Nutrition ESPEN* (IF = 2.9) and *Nutrition in Clinical Practice* (IF = 2.1) had impact factors below 3.000.

### 3.7 Analysis of highly cited articles

The articles listed in Table 6 are ranked in descending order based on total citation count. The top 10 most cited studies were primarily published between 2019 and 2022. Leading the list by a significant margin is the article titled “GLIM criteria for the diagnosis of malnutrition – A consensus report from the global clinical nutrition community” (9) (IF = 6.6), which has received 2,807 citations. It is followed by “Malnutrition in Older Adults—Recent Advances and Remaining Challenges” (34) (IF = 4.8; 334 citations), and “Guidance for assessment of the muscle mass phenotypic criterion for the Global Leadership Initiative on Malnutrition (GLIM) diagnosis of malnutrition” (35) (IF = 6.6; 166 citations).

**TABLE 6:** Top 10 Most Cited GLIM-Related Articles (2018–2024).

Rank	Year	Article	IF (2023)	Total citation
1	2019	Cederholm T, 2019, J Cachexia Sarcopenia Muscle	6.6	2,807
2	2021	Norman K, 2021, Nutrients	4.8	334
3	2022	Barazzoni R, 2022, Clinical Nutrition	6.6	166
4	2022	Sayer AA, 2022, Age and Aging	6.0	162
5	2021	Zhang X, 2021, Clinical Nutrition	6.6	152
6	2019	Beaudart C, 2019, Nutrients	4.8	151
7	2020	Bedock D, 2020, Clinical Nutrition ESPEN	2.9	149
8	2020	De Van Der Schueren MAE, 2020, Clinical Nutrition	6.6	145
9	2019	Contreras-Bolívar V, 2019, Nutrients	4.8	144
10	2020	De Groot LM, 2020, Nutrients	4.8	131

IF(2023), 2023 Journal Impact Factor.

### 3.8 GCS analysis of highly influential publications

**FIGURE 4:**
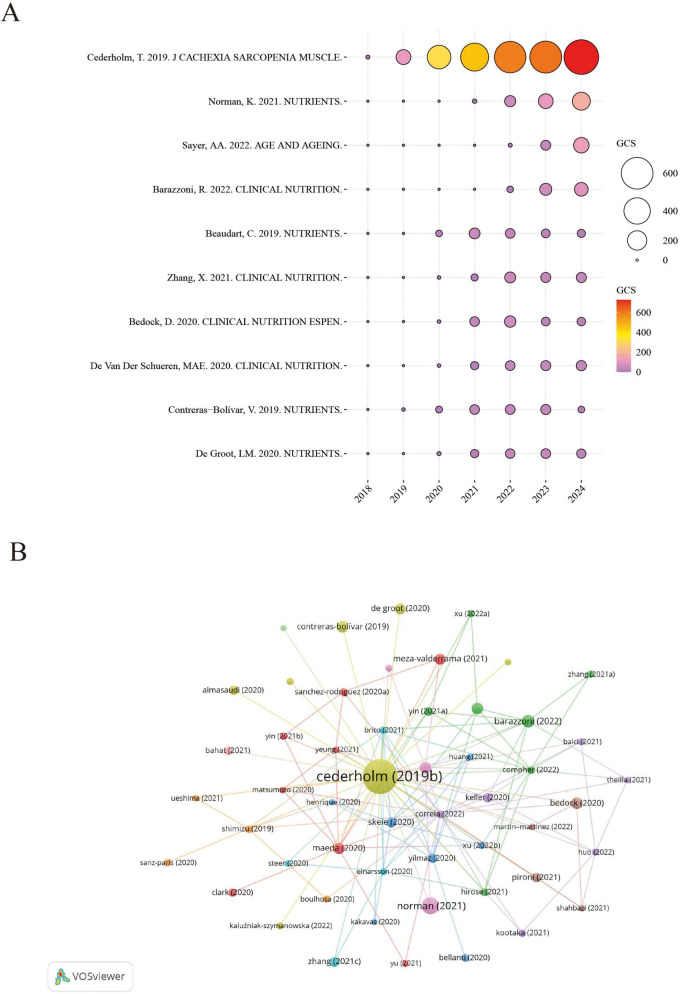
Citation impact and co-citation analysis of highly influential GLIM-related references. **(A)** Annual GCS of the top-cited references. Bubble size and color reflect GCS values, with larger and redder circles indicating higher global citation impact. **(B)** Reference co-citation network generated from 729 GLIM-related publications. A minimum co-citation threshold of 40 was applied, resulting in the inclusion of 63 references. Node size represents co-citation frequency, node color indicates cluster grouping, and line thickness reflects co-citation strength. GCS (Global Citation Score): Total citation count extracted from Scopus database.

Figure 4A presents a bubble chart illustrating the annual GCS of highly cited references in the GLIM research field, offering insight into their academic influence and citation trends over time. Notably, the 2019 GLIM consensus report by Cederholm et al. recorded a peak GCS of 727 in 2024, with a total citation count of 2,927, significantly outpacing other publications. This underscores its pivotal role in the development and validation of the GLIM criteria. Since its publication, the paper has shown a steady increase in citations, reaching its highest impact in 2024, further reflecting its importance in clinical nutrition research. In addition to Cederholm’s work, the 2021 review by Norman K., published in *Nutrients*, also showed a high GCS in 2024, highlighting the growing attention toward malnutrition in older adults. Other studies—such as Sayer et al. (36) in *Age and Aging*, Barazzoni et al. (35) in *Clinical Nutrition*, and Beaudart et al. (37) in *Nutrients*—have also maintained substantial academic influence in recent years.

Based on the reference co-citation network (Figure 4B), key publications in the GLIM field and their interrelationships were identified. The visualization highlights clusters of frequently co-cited studies, reflecting thematic connections and intellectual structure within the research landscape.

Overall, Figure 4A reveals distinct citation trajectories among highly cited GLIM-related publications, with the 2019 consensus report by Cederholm et al. standing out as the most influential. Meanwhile, several other core papers have continued to attract attention.

### 3.9 Research hotspot analysis

**FIGURE 5:**
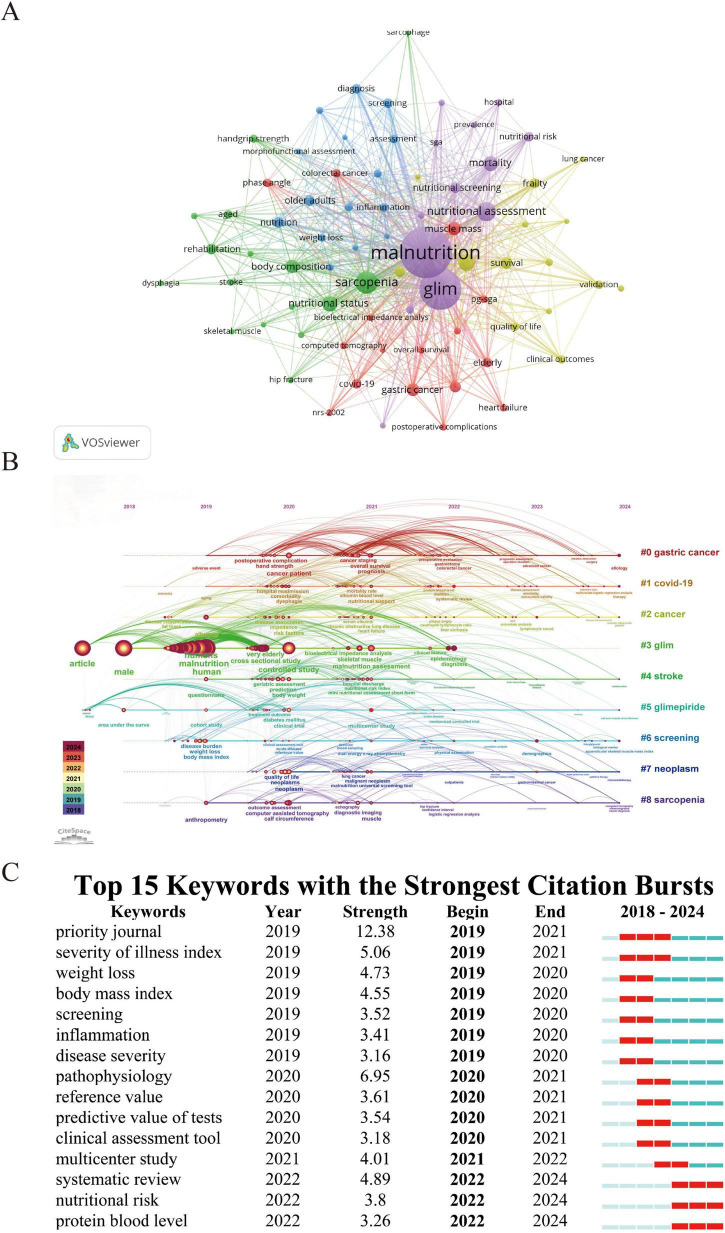
Keyword mapping and research trends related to GLIM. **(A)** Co-occurrence network of 74 keywords that appeared more than six times, grouped into five clusters and visualized in different colors: Red, yellow, blue, green, and purple. Node size reflects keyword frequency. **(B)** Timeline view of keyword clustering analysis, showing the temporal distribution and evolution of research themes across the period 2018–2024. **(C)** Top 15 keywords with the strongest citation bursts. The “Begin” and “End” years indicate the time period during which each keyword had high citation intensity. Light green indicates years before the keyword emerged, dark green indicates periods of low influence, and red indicates years of high citation burst intensity.

Beyond the pre-defined search terms, author keyword clustering was conducted on 729 publications using VOSviewer and CiteSpace. As shown in Figure 5A, five major clusters were identified: Cluster 1 (purple) focuses on the standardized GLIM assessment system and nutritional screening tools; Cluster 2 (green) relates to muscle health and rehabilitation interventions; Cluster 3 (red) highlights the nutritional risk characteristics of elderly and chronically ill patients; Cluster 4 (blue) emphasizes nutritional diagnosis and inflammatory biomarkers in older populations; and Cluster 5 (yellow) centers on prognosis and clinical outcomes in cancer patients. High-frequency keywords such as “diagnosis,” “inflammation,” and “survival” suggest that current research is concentrated on the clinical translation of the GLIM framework and the interactions between malnutrition, inflammation, and disease progression.

As illustrated in Figure 5B, keywords like “malnutrition,” “screening,” “diagnosis,” and “bioelectrical impedance analysis” have remained central themes in GLIM-related research over time. Meanwhile, Figure 5C reveals that in the past 2 y, emerging hotspots include “systematic review,” “nutritional risk,” “protein blood level,” and “multicenter study,” indicating a recent shift toward evidence synthesis, personalized nutritional assessment, and clinical validation within the GLIM research landscape.

## 4 Discussion

### 4.1 Global publication trends and regional differences

This study represents the first systematic bibliometric analysis of research related to the GLIM framework. Using data retrieved from the Scopus database, combined with visualization tools such as VOSviewer and CiteSpace, we systematically mapped and analyzed research trends and thematic hotspots in the GLIM literature. A total of 729 publications, including original articles and reviews published between 2018 and 2024, were identified. Although annual publication counts have shown some fluctuations in recent years, the polynomial regression model demonstrated a steadily increasing trend, with a notable surge in research activity observed after 2020. This growth pattern suggests that GLIM has attracted increasing scholarly attention.

As shown in Figure 2A, the global distribution of GLIM-related publications from 2018 to 2024 reveals that China ranked first with 191 articles (26.20%), followed by Spain (93 articles, 12.76%) and Japan (92 articles, 12.62%). However, despite China’s leading position in Np, countries such as Sweden and the United States demonstrated greater influence in terms of Nc and H-index when considering national, institutional, and author-level metrics. For example, Uppsala University in Sweden ranked first among institutions, with an Nc of 4,024 and an H-index of 19, far surpassing Chinese institutions such as Capital Medical University (Nc = 1,003; H-index = 17). At the author level, Cederholm, T., also from Sweden, led with 3,937 citations and an H-index of 18. In contrast, although Chinese scholars occupied five of the top ten positions in terms of Np, their average Nc values were substantially lower. For instance, Shi, H. had 422 citations, significantly fewer than his European and American counterparts. This disparity may reflect differences in research focus: The Swedish team, including Cederholm et al., concentrated on the theoretical development and international consensus of the GLIM framework (e.g., the 2019 consensus report with 2,807 citations), while many Chinese studies emphasized regional clinical data analyses. To enhance global academic influence, Chinese researchers are encouraged to strengthen international collaboration, improve theoretical innovation, and publish in high-impact journals. Although Spain demonstrated a relatively high H-index (22), its Nc (1,790) remained substantially lower than countries with similar Np, suggesting a need to further enhance the quality and impact of its research output.

### 4.2 Institutional and author-level contributions

Among the top 10 contributing institutions, six are based in China, highlighting the country’s strong academic presence in this field. The institution with the highest publication volume is Capital Medical University (36 publications, 1,003 citations), followed by Uppsala University in Sweden (35 publications, 4,024 citations) and Beijing Shijitan Hospital in China (33 publications, 979 citations). Regarding author contributions, the top three most productive scholars are Cederholm, T. (Sweden; 30 publications, 3,937 citations), Shi, H. (China; 22 publications, 422 citations), and Correia, M.I.T.D. (Brazil; 19 publications, 3,667 citations). These researchers have demonstrated both high productivity and academic influence, underscoring their leadership roles in the field. Greater attention should be paid to their work, as it often reflects the forefront of GLIM research and can guide future investigations. Notably, a highly valuable collaborative network has formed among these core scholars. Collaborative studies between Cederholm and Correia (2019–2020) laid the theoretical foundation for the GLIM criteria. Their 2019 consensus report was the first to systematically propose the diagnostic standards for GLIM, aiming to standardize the definition, assessment process, and diagnostic framework of malnutrition (9). This landmark paper, endorsed by the global clinical nutrition community, provides unified diagnostic criteria applicable across various healthcare settings and serves as a foundation for future clinical research and guideline development. The 2020 follow-up study established operational standards for validating the GLIM criteria. It proposed a hybrid validation strategy combining retrospective cohort analyses and prospective clinical studies and incorporated machine learning techniques to optimize threshold selection, ensuring the diagnostic criteria’s applicability and reliability across populations and clinical scenarios (28). In 2021, a collaboration between Cederholm and Shi promoted the clinical translation of GLIM in oncology nutrition. Three pivotal studies collectively demonstrated the multidimensional utility of GLIM: First, a study in older adult cancer patients (*n* = 1,192) showed that GLIM-defined malnutrition independently predicted mortality risk (HR = 1.71), and a nomogram integrating clinical variables achieved accurate prognostic stratification (C-index = 0.82) (29). Second, the development of the Scored-GLIM system (*n* = 3,547) quantified the prognostic weight of core indicators such as involuntary weight loss (HR = 1.82), significantly improving 1-year survival prediction (C-index = 0.62) and demonstrating superior net clinical benefit compared to traditional GLIM assessments (30). Third, a multicenter cohort study involving 3,777 patients confirmed the moderate diagnostic agreement between GLIM and the sPG-SGA (κ = 0.54), with sensitivity and specificity of 70.5 and 88.3%, respectively, supporting GLIM as a valid and efficient alternative to the sPG-SGA (31). These findings provide robust evidence for standardized GLIM application in oncology settings. Further collaboration among Cederholm, Shi, and Correia addressed key methodological challenges in GLIM implementation. A 2022 guideline paper systematically defined muscle mass assessment methods, recommending imaging techniques (e.g., DXA, CT) as the preferred options while also offering anthropometry-based alternatives for resource-limited settings (32). In 2024, a new consensus based on the modified Delphi approach achieved a high level of expert agreement and introduced a composite diagnostic framework that integrates clinical evaluation, laboratory testing, and underlying disease analysis to enhance the global applicability of GLIM criteria (33). These methodological advances have laid a strong foundation for the standardized implementation of GLIM across diverse healthcare settings.

### 4.3 Journal distribution and dissemination pathways

It is noteworthy that eight of the top 10 most productive journals in GLIM-related research have relatively high impact factors (IFs). Journals such as *Nutrients*, *Clinical Nutrition, Frontiers in Nutrition*, and *the Journal of Parenteral and Enteral Nutrition (JPEN)* have made significant contributions to the field. This phenomenon may be partly attributed to the relatively high IFs of these journals, which enhance their academic appeal. More importantly, their research scopes are highly aligned with the scientific focus of the GLIM criteria, particularly in areas such as clinical nutrition, metabolic disorders, geriatrics, and dietary interventions. This alignment increases the relevance and visibility of GLIM-related studies within these journals, making them preferred publication venues for researchers in the field. To elaborate, *Nutrients* is an open-access journal that covers a broad range of topics in nutritional science, including basic and applied nutrition, dietary patterns, metabolic health, and chronic disease prevention. It is dedicated to promoting the role of dietary interventions in disease management. *Clinical Nutrition*, the official journal of *the European Society for Clinical Nutrition and Metabolism (ESPEN)*, focuses on the latest advances in clinical nutrition, especially in the areas of malnutrition, metabolic disorders, and nutritional support strategies, and has played a central role in the development and dissemination of the GLIM criteria. *Frontiers in Nutrition* mainly publishes high-quality research in food science, dietary patterns, nutritional metabolism, and health promotion, emphasizing the integration of basic research and clinical application. JPEN, a leading journal in the field of parenteral and enteral nutrition, concentrates on clinical nutrition therapies, nutrition support techniques, and their application in disease management. It holds substantial influence in the safety and efficacy assessment of nutritional support strategies. Collectively, the strong alignment between these journals’ scopes and the core themes of GLIM research, along with their academic visibility and relatively efficient peer-review process, has contributed to their status as preferred publication venues for GLIM-related studies.

Among the top 10 most cited articles, the study by Cederholm T. recorded the highest GCS in 2024 (727). This landmark publication introduced the GLIM diagnostic framework, which has since become the most cited reference in the field. The study established standardized criteria for the identification, assessment, and stratification of malnutrition across global clinical settings, effectively addressing the long-standing gap in unified nutritional care guidelines (9). Subsequent research has further validated and expanded the application of the GLIM criteria. Norman et al. provided a comprehensive review of malnutrition in older adults, detailing its multifactorial etiology—including age-related physiological changes, comorbidities, and chronic inflammationon of the GLIM criteria. Norman et al. provided a comprehensive revie (34). Barazzoni et al. contributed significant methodological advancements through a consensus guideline, proposing standardized approaches for muscle mass assessment using DXA, CT, and BIA, while offering anthropometric alternatives for low-resource environments (35). Through the Global Leadership Initiative on Sarcopenia (GLIS), Sayer et al. refined the diagnostic model of sarcopenia, highlighting the importance of muscle functione functionGLIS), Sayer et al. reometric alternativeto GLIM-related frameworks (36). Zhang et al. confirmed the clinical utility of the GLIM criteria in elderly cancer patients, demonstrating its value in diagnosing malnutrition and predicting survival outcomes. They also developed a nomogram based on GLIM indicators with strong prognostic performance (29). Beaudart et al., analyzing 4-y data from the SarcoPhAge cohort, revealed a significant association between malnutrition and the incidence of sarcopenia and severe sarcopenia, underscoring the need for early nutritional intervention (37). In the context of the COVID-19 pandemic, Bedock et al. found a malnutrition prevalence of 42.1% among hospitalized patients and identified hypoalbuminemia as a predictor of ICU admission, reinforcing the clinical necessity of nutritional screening (38). De van der Schueren et al. systematically evaluated the feasibility and validation strategies of the GLIM criteria, advocating for consensus-based diagnostic frameworks and recommending the use of large-scale datasets and machine learning to improve reliability and adaptability across populations (28). Contreras-Bolontrerass populations adaparning mmending the use of largealidation strategies 6-month mortality in hospitalized cancer patients, reinforcing the role of functional assessment in malnutrition diagnosis (39). Lastly, De Groot et al. compared the performance of the PG-SGA SF and GLIM criteria in ambulatory cancer care, showing that PG-SGA SF had higher sensitivity and specificity, while GLIM effectively predicted 1-year mortality, although the addition of HGS did not significantly enhance diagnostic accuracy (40).

### 4.4 Thematic evolution and emerging research frontiers

Keyword clustering analysis reveals that GLIM-related research primarily revolves around five thematic clusters, reflecting the key research focuses and developmental trends in the field. The purple cluster includes keywords such as malnutrition, GLIM, nutritional assessment, mortality, and nutritional screening, emphasizing the application of the GLIM criteria in clinical malnutrition evaluation and screening. The green cluster features sarcopenia, body composition, nutritional status, and rehabilitation, underscoring the relevance of GLIM in sarcopenia research and body composition analysis. The red cluster, represented by terms like PG-SGA, elderly, gastric cancer, COVID-19, and heart failure, reflects the widespread use of GLIM in assessing nutritional risk among older adults and patients with chronic or acute illnesses. The blue cluster centers on nutrition, older adults, diagnosis, inflammation, and screening, highlighting the value of GLIM in geriatric nutrition assessment, inflammation-related studies, epidemiological research, and disease screening. The yellow cluster includes cancer, survival, frailty, validation, clinical outcomes, and quality of life, indicating a growing focus on prognosis, outcome optimization, and frailty assessment in cancer populations. CiteSpaceil timeline analysis further reveals the dynamic evolution of research topics within the GLIM domain. Between 2018 and 2024, keywords such as malnutrition, sarcopenia, and screening appeared consistently across multiple time periods, suggesting that these themes have remained long-standing focal points of GLIM research. Since 2022, however, the research focus has shifted noticeably toward clinical validation and personalized nutritional assessment. Burst keyword analysis shows that from 2019 to 2021, GLIM research primarily focused on weight loss, body mass index (BMI), and screening, highlighting efforts to define malnutrition and establish diagnostic standards. In contrast, between 2022 and 2024, systematic review (burst strength: 4.89) and multicenter study (burst strength: 4.01) emerged as major hotspots, indicating a transition toward evidence synthesis and clinical validation. Moreover, nutritional risk and protein blood level have become newly emerging burst keywords, suggesting that biomarker detection and precision nutritional assessment may be key directions for future research. Looking ahead, GLIM research is expected to continue evolving toward precision nutritional interventions, incorporating biomarker profiling, genomics, and machine learning to enhance individualized diagnosis and nutrition management strategies. In parallel, strengthening multicenter clinical validation will be critical to improving the applicability and reliability of the GLIM criteria across diverse populations.

Based on the keyword timeline and burst analysis, we identified three representative research themesnd burst analysis, we identified a across diverse populations.al validatnered increasing attention and demonstrated a rising trend over the past 2 years. The following sections provide an in-depth discussion of these emerging hotspots to further elucidate the developmental trajectory and potential directions of current research frontiers.

#### 4.4.1 Gastric cancer

Gastric cancer is a globally prevalent malignancy with poor prognosis, and malnutrition is common among patients, with reported prevalence ranging from 19 to 70.6% (41). Malnutrition in this population is closely associated with postoperative complications, reduced survival rates, and impaired quality of life. In recent years, GLIM has gained increasing attention in the nutritional management of gastric cancer due to its standardized diagnostic approach. Clinical studies have shown that severe malnutrition, as defined by GLIM, is an independent risk factor for postoperative complications (Clavien-Dindo grade ≥ II, OR: 1.339lications (Claviendent risk facshown that severe) (42). However, the prognostic implications of moderate malnutrition remain inconsistent, with some studies reporting a significant reduction in OS, while others failed to confirm such associations (43). Several studies conducted in China have highlighted diagnostic discrepancies between GLIM and PG-SGA. PG-SGA is widely regarded as the s widely regarded cted in China have higent. A study by Qin et al. (44) reported malnutrition prevalence rates of 65% using GLIM and 74.2% using PG-SGA, suggesting that GLIM may underestimate the risk of sarcopenia. Similarly, studies by Xu et al. (45) and Zheng et al. (46) reported moderate agreement between the two tools (Cohen’s κ = 0.483–0.548). Zheng et al. (46) further demonstrated that malnutrition prevalence based on GLIM was 68.81%, compared to 76.76% with PG-SGA. Moreover, PG-SGA exhibited superior sensitivity and specificity in identifying malnutrition. Recent research efforts have focused on optimizing the clinical applicability of GLIM in gastric cancer patients. Various studies have validated the consistency between muscle mass assessment tools, such as CT and HGS, in the diagnosis of malnutrition. For example, Huang et al. reported a high agreement between HGS-based GLIM classification and CT-derived SMI, with a Cohen and CT malnutrition. For example, Huang et aHGS as a reliable alternative in resource-limited settings (47). Zhou et al. also confirmed the feasibility and diagnostic accuracy of using HGS for muscle mass assessment within the GLIM framework (48). Additionally, combining VAT evaluation with GLIM has been shown to enhance predictive accuracy. Zhang et al. found that incorporating low VAT improved the AUC from 0.78 to 0.83 in predicting postoperative adverse outcomes and long-term survival, suggesting that VAT assessment adds prognostic value (49). Beyond conventional tools, AI, particularly deep learning models based on CT imaging, has been introduced for early detection of malnutrition. These models, which integrate clinical data such as BMI and lymphocyte counts, demonstrated high accuracy (AUC = 0.857) and offer promising potential for personalized nutritional intervention (50). Despite the clinical potential of GLIM in gastric cancer, several challenges hinder its broader implementation. First, differences in muscle mass thresholds between East Asian and Western populations (e.g., SMI < 40.8 cm^2^/m^2^ in East Asian men vs. < 52.4 cm^2^/m^2^ in Western men) may lead to diagnostic bias, underscoring the need for population-specific adaptations (41). Second, dynamic postoperative nutritional monitoring (e.g., phase angle and continuous glucose monitoring) has revealed significant associations between nocturnal hypoglycemia (TBR > 20%) and severe malnutrition defined by GLIM. In one study, the incidence of nocturnal hypoglycemia was notably higher in severely malnourished patients compared to those with normal or moderate nutritional status (TBR: 41.1 vs. 30.6%, *P* = 0.034). However, such biomarkers have not yet been incorporated into the current GLIM framework (51). To further advance the application of GLIM in gastric cancer nutrition management, future studies should move beyond traditional assessment methods by integrating real-time monitoring, multi-source data, and AI technologies. These efforts must also account for population-specific characteristics and practical implementation feasibility, with the ultimate goal of enabling more precise and personalized nutritional interventions.

#### 4.4.2 Protein blood level

Protein blood levels serve as important biomarkers for assessing malnutrition and inflammatory status, and occupy a critical role in the GLIM framework. They are widely used in clinical practice to evaluate nutritional status and predict disease prognosis. Among them, serum albumin and pre-albumin are the most frequently tested indicators. Low levels of these proteins not only suggest malnutrition but are also closely associated with various adverse clinical outcomes. In hospitalized cancer patients, Contreras-Bolncer patients, Contrerasteins not only suggest malnutrition but are also closely asso-defined malnutrition and independently predicted 6-month mortality (OR = 2.72, *p* = 0.004) (39). Pre-albumin, as a more sensitive marker of short-term nutritional changes, has been shown to correlate with decreased muscle mass and functional decline. In patients with systemic sclerosis, low pre-albumin levels were often accompanied by reduced handgrip strength and fat-free mass, highlighting its potential utility in muscle function assessment (52).

However, in inflammatory states, reliance on serum proteins—especially albumin and prealbumin—for nutritional assessment is problematic. As negative acute-phase reactants, their hepatic synthesis is downregulated and capillary permeability increases under systemic inflammation, leading to reduced serum levels that do not reflect actual nutritional status (7). Although they often correlate with adverse clinical outcomes, such associations mainly indicate disease severity and inflammatory burden rather than true nutritional reserves. Accordingly, GLIM no longer includes serum proteins as diagnostic criteria due to their limited specificity and sensitivity (53). Supporting this concern, a recent study found that nearly 23% of patients identified as malnourished still had normal pre-operative albumin concentrations (54).

Several studies have explored multidimensional models that integrate protein biomarkers with other nutritional and inflammatory indicators to improve clinical utility. Olpe et al. reported that while albumin alone had modest prognostic value (AUC = 0.62), combining it with muscle mass and function parameters in a multidimensional model improved prediction accuracy (AUC = 0.71) (55). The integration of serum proteins with inflammatory markers also shows significant clinical promise. Pourhassan et al. suggested that C-reactive protein (CRP) ≥ 3.0 mg/dL could serve as a risk threshold for inflammation-associated malnutrition, strongly linked to recent reductions in food intake (OR = 1.61) (56). Shi et al. further demonstrated a positive correlation between serum GDF15 and CRP (*r* = 0.318, *p* < 0.001). When elevated GDF15 was combined with low albumin (< 36.15 g/L), the model’s predictive accuracy for malnutrition significantly improved (AUC = 0.935), outperforming single indicators (57). In prognostic evaluation, protein biomarkers also play a pivotal role. Liu et al. developed a nomogram based on GLIM to predict postoperative complications and confirmed that low albumin was an independent risk factor following cardiac surgery (OR = 1.66, AUC = 0.72) (58). In lung cancer patients, Yin et al. found that combining low albumin (< 35 g/L) with decreased muscle mass effectively distinguished prognostic subgroups, with a median survival gap of up to 9 months (*P* < 0.001), supporting its use in personalized nutrition-prognosis models (59).

In conclusion, protein blood levels not only aid in diagnosing malnutrition within the GLIM framework but also serve as valuable tools for assessing inflammation, prognosis, and response to nutritional interventions. Future research should aim to standardize measurement methods and explore the combined use of emerging biomarkers such as GDF15, thereby enhancing the precision and clinical utility of GLIM.

#### 4.4.3 Nutritional risk

In the diagnostic process outlined by GLIM, the first and most important step is to screen patients for nutritional risk. This step helps identify people who may need further evaluation to confirm if they are malnourished. However, in real clinical settings, the tools used for screening are quite varied, and their accuracy can differ depending on the population. This makes it hard to apply the GLIM process in a consistent and standardized way. A review by Correia et al. showed that only 57% of studies actually followed GLIMhowed that onlytwo-step approachctually follirst, then diagnosis—suggesting that many real-world applications still fall short (60). Similarly, research by MacDonell et al. found that in a diverse elderly population in New Zealand, the SCREEN-II tool predicted 5-year death rates better than the GLIM criteria for Māori individuals. This highlights the need to tailor screening tools to specific ethnic groups and local populations (61). Once the importance and challenges of identifying nutritional risk became clear, researchers started paying more attention to which screening tools work best in different situations. For example, in a large study, Tan et al. found that using GLIM together with the MNA-SF tool gave the most accurate predictions of infection and wound problems after abdominal cancer surgery (62). However, Kutnik et al. noted that although about 20% of elective surgery patients were found to be at nutritional risk, very few of them actually received any nutritional treatment, pointing to a gap between identifying risk and taking action (63). To make screening more accurate and practical, some studies have looked at objective physical indicators. For instance, Fu et al. found that measuring calf circumference is a useful and easy way to estimate muscle loss in Asian patients with gastric cancer (64). Another study by Muñoz-Redondo et al. showed that patients with a phase angle (PhA) of ≤ 4.85 had a much higher chance of being malnourished (odds ratio = 3.53), suggesting that PhA could be a helpful clinical tool for identifying those at risk (65). In addition to being a first step in diagnosis, nutritional risk screening is now also used to help plan treatment strategies. In a follow-up analysis of the EFFORT trial, Kaegi-Braun et al. found that hospitalized patients who were both GLIM-positive and at nutritional risk showed clear improvement when they received personalized nutrition support (odds ratio = 0.69). However, patients who were not at risk didnwedseem to benefit, showing that risk screening can help doctors decide who is most likely to benefit from treatment (66). To make screening even more efficient, researchers have started using artificial intelligence (AI). Wu et al. created machine learning models for patients with colorectal cancer, which could accurately detect GLIM-defined malnutrition even without knowing the patient’s weight loss. Their random forest model had a strong predictive performance (AUC = 0.83), and they also developed an online prediction tool. This shows that AI can help improve how we identify patients at risk, even when clinical data is incomplete, making the GLIM process more useful and accurate in real-world settings (67).

Overall, the emergence of gastric cancer, protein blood biomarkers, and nutritional risk as leading research hotspots underscores a critical transition in GLIM-related studiescfrom establishing diagnostic criteria toward optimizing their clinical applicability and predictive utility. Despite significant progress, challenges remain in achieving diagnostic consistency across populations, integrating objective biomarkers into routine practice, and tailoring nutritional assessment tools to diverse clinical contexts. Future research should prioritize refining GLIM-based models through multicenter validation, biomarker standardization, and deeper investigation into the mechanistic links between malnutrition and disease progression. Such efforts will not only enhance the diagnostic precision of the GLIM framework but also support its broader adoption in global nutritional care systems.

### 4.5 Strengths and limitations of the study

This study has several notable strengths. First, it represents the first systematic bibliometric analysis of GLIM-related research, addressing a gap in the quantitative assessment of this field. The findings provide comprehensive and visualized insights for researchers interested in the evolution of malnutrition diagnostic standards. Second, we employed three mainstream bibliometric toolslVOSviewer, CiteSpace, and the Bioinformatics online platform. VOSviewer and CiteSpace are well-established tools in medical bibliometric research and greatly enhance the scientific rigor and objectivity of our analysis. Third, compared with traditional narrative reviews, bibliometric analysis allows for a more systematic and traceable exploration of research hotspots and frontier trends in GLIM research, while also identifying key authors, institutions, and highly influential publications. However, this study also has some limitations. First, all data were extracted solely from the Scopus database, and studies from other databases may have been missed. Second, only English-language publications were included, which may underestimate the contribution of non-English research.

## 5 Conclusion

This study conducted a systematic bibliometric analysis of research on the GLIM criteria from 2018 to 2024. The findings show that since the introduction of the GLIM standard, related research has developed rapidly, with the number of publications increasing steadily and accelerating notably after 2020—reflecting growing academic interest in this field. China and Sweden emerged as leading contributors, with China taking the lead in publication volume, while Sweden demonstrated strong performance in highly cited papers and research impact. Despite the overall research activity, international institutional collaboration remains an area for improvement. Clinical *Nutrition* and *Nutrients* were identified as the most influential journals in this domain, leading in both publication output and academic dissemination. In addition, this study used keyword co-occurrence and burst analysis to uncover research hotspots and their evolution, offering valuable insights and directions for future investigations in the GLIM field.

## Data Availability

The dataset analyzed in this study was retrieved from the Scopus database (https://www.scopus.com/) using institutional access. Due to licensing restrictions, the raw dataset cannot be made publicly available in its entirety. However, the original bibliographic data file (in.csv format) used for VOSviewer and CiteSpace analysis has been provided as Supplementary material. Additional information is available from the corresponding authors upon reasonable request.

## Footnotes

1
https://www.bibliometrix.org

2
https://www.bioinformatics.com.cn
